# Progress in Sol–Gel-Derived Phenolic Aerogels: Control of Network Topology, Drying Technologies, and Functional Modification

**DOI:** 10.3390/polym18162029

**Published:** 2026-08-21

**Authors:** Hongwei Yang, Zongyi Deng, Minxian Shi, Zhixiong Huang

**Affiliations:** 1 School of Materials Science and Engineering, Wuhan University of Technology, Wuhan 430070, China; 2 Sanya Science and Education Innovation Park, Wuhan University of Technology, Sanya 572000, China; 3 Hubei Longzhong Laboratory, Xiangyang 441000, China

**Keywords:** phenolic aerogels, sol–gel, microstructure regulation, drying processes, thermal protection, functional applications

## Abstract

Phenolic aerogels, owing to their low density, high char yield, large specific surface area, and well-defined three-dimensional topological networks, hold considerable promise for applications in extreme thermal protection and multifunctional material systems. The sol–gel process, a cornerstone methodology for constructing the three-dimensional nanoporous architecture of these materials, critically governs the resulting microstructural topology and macroscopic performance through its reaction kinetics, phase-separation behavior, and drying dynamics. This review systematically surveys recent advances in the sol–gel synthesis of phenolic aerogels, focusing on the polycondensation mechanisms operative under acidic and basic catalytic conditions, nucleation-and-growth kinetics, and strategies for tailoring multiscale pore structures. It further provides a comparative analysis of interfacial regulation mechanisms for capillary-stress elimination across supercritical drying, freeze-drying, and ambient-pressure drying routes. We also dissect the structure–property relationships underpinning Knudsen-effect-mediated gaseous thermal insulation, multi-scale hybrid network toughening, and inorganic phase-transition-induced in situ ceramization for thermal protection, demonstrating the synergistic optimization of thermal insulation, structural load-bearing, and ablation resistance. Finally, we summarise current applications in extreme thermal protection, environmental adsorption, electromagnetic interference shielding, and electrochemical energy storage and highlight future directions towards green, scalable manufacturing and intelligent materials design.

## 1. Introduction

**Figure 1:**
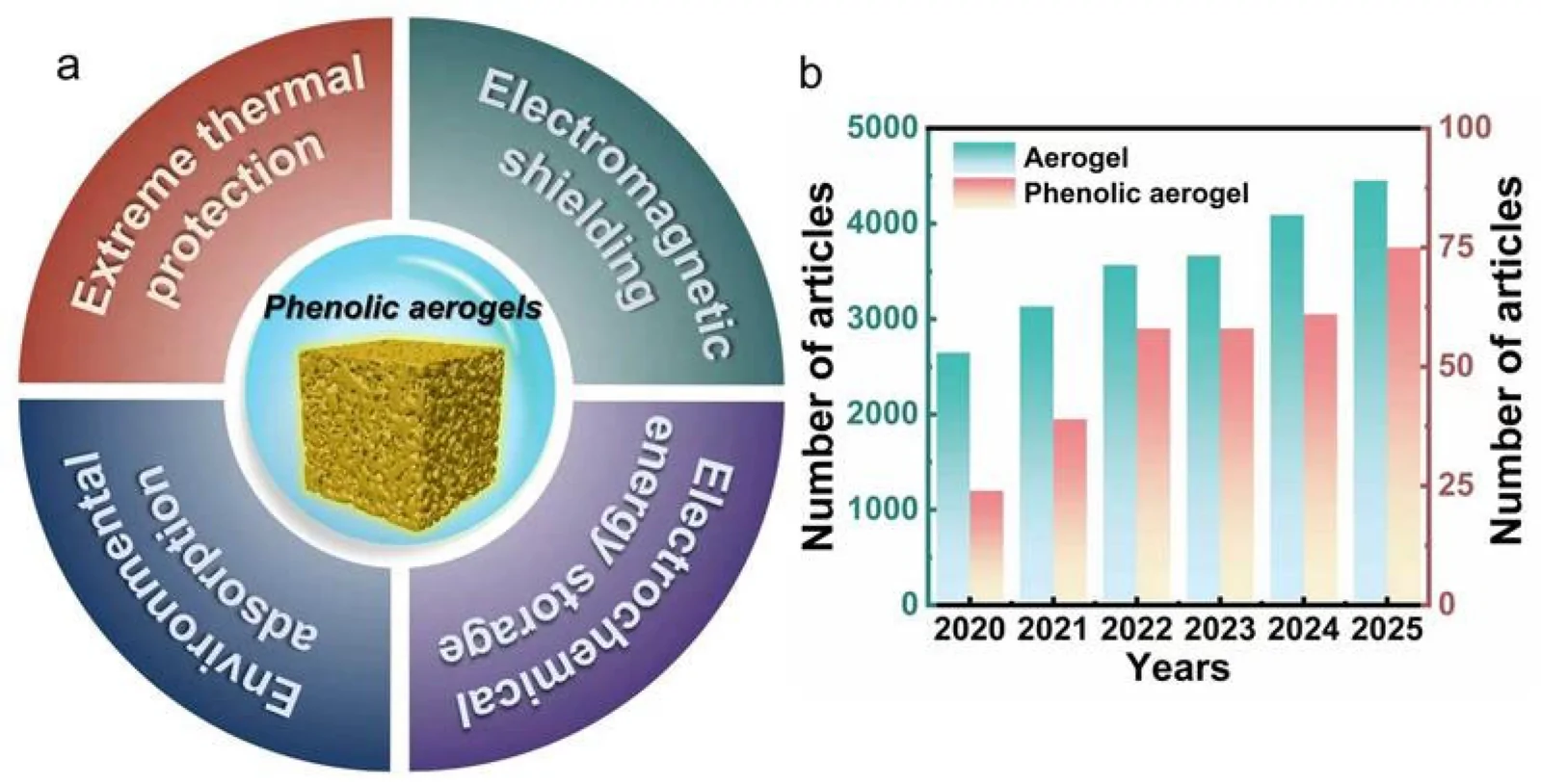
(**a**) Applications of phenolic aerogels and (**b**) recent publication trends for aerogels and phenolic aerogels. Data are from the Web of Science database, retrieved as of July 2026.

Driven by the ever-increasing demand for lightweight, high-performance thermal insulation materials in extreme service environments—such as aerospace, energy storage, and high-temperature thermal protection—porous material systems, particularly aerogels, have attracted extensive attention owing to their ultralow density and extremely low thermal conductivity [1,2,3,4]. Since Pekala first successfully synthesized organic aerogels in the late 1980s, research on polymer aerogels and their derived carbonaceous counterparts has gained remarkable momentum [5,6,7,8]. Phenolic aerogels, as typical representatives of organic aerogels and nanoporous functional materials with a three-dimensional network structure, combine excellent thermal stability, high char yield, and favorable molecular designability, thereby showing great potential for applications in extreme thermal protection, environmental adsorption, electromagnetic shielding, and electrochemical energy storage (Figure 1a) [9,10,11].

The sol–gel process is central to the fabrication of high-performance phenolic aerogels, enabling controlled evolution from the molecular scale to the macroscopic pore structure through the coupled effects of addition–condensation reaction kinetics, phase separation, and gelation [12,13,14]. Notwithstanding the significant progress achieved in gel chemistry control and microstructure design, this process still faces systematic challenges towards industrialisation: intrinsic brittleness arising from the crosslinked network compromises mechanical reliability; the high cost of supercritical drying and the interfacial mechanisms of ambient-pressure drying remain to be overcome; and the integrated design of biomass substitution and multifunctional integration presents a significant challenge [15,16,17,18]. Therefore, grounded in the sol–gel reaction mechanism, achieving synergistic regulation of pore structure, crosslinking density, and multiscale architecture has emerged as a critical research direction in this field.

A survey of Web of Science (WoS) publications over the past six years (2020–2025) reveals that research articles on the structural optimization and property enhancement of phenolic aerogels have grown explosively (Figure 1b), driven by the reciprocal reinforcement among demand pull, technological advancement, performance improvement, and application expansion. Researchers have proposed various regulatory strategies, including molecular structure design, polymerization-induced phase separation modulation, nanofiller incorporation, and drying process optimization [19,20,21,22,23]. Effective modulation of the network structure has been achieved across multiple length scales, enabling a marked improvement in mechanical strength and thermal stability while preserving the intrinsically low-density feature of the materials. In particular, the introduction of inorganic nanophases or carbon-based reinforcing phases has not only improved the collapse resistance of the skeletal framework but also further extended its application potential in high-temperature thermal insulation and multifunctional composite systems. With this in mind, our group has conducted in-depth investigations on high-performance phenolic aerogels in our previous work [24,25,26,27].

In this context, Ding et al. established a fundamental knowledge framework for phenolic aerogels that spans their preparation, modification, and application, thereby offering a systematic overview that has advanced the field; however, their discussion remains relatively superficial regarding the advanced chemical modification mechanisms and interfacial heat-transfer decoupling under extreme thermal protection conditions [14]. In contrast, Ma et al. have further expanded their work to molecular engineering, multi-element coupling, and the design of multifunctional thermal protection systems, reflecting the evolutionary trend of phenolic aerogels from conventional thermal insulation toward high-performance, multifunctional, and lightweight thermal protection materials. However, their focus remains primarily on aerospace thermal protection, with relatively limited coverage of fundamental synthesis processes and other application areas [28]. In this context, this review summarizes the progress since 2020 in the preparation of phenolic aerogels via the sol–gel method, with a focus on reaction mechanisms, kinetic regulation, pore-structure formation, and the impact of drying processes on the microstructure. It further outlines the principal strategies and emerging trends in structural control and performance optimization and finally surveys their current application status in extreme thermal protection, environmental adsorption, electromagnetic shielding, and electrochemical energy storage. Finally, we discuss the critical challenges and future directions for functional applications to provide a theoretical foundation and practical guidance for the design and controlled synthesis of high-performance phenolic aerogels.

## 2. Sol–Gel Reaction Mechanisms and Microstructure Regulation

The three-dimensional nanoporous network topology of phenolic aerogels directly governs their macroscopic properties, including density, specific surface area, mechanical strength, and thermal conductivity [29,30,31]. This structure is fundamentally determined by the kinetic pathways of cascade chemical reactions among precursor molecules during the sol–gel transition [32]. Therefore, this chapter discusses the reaction mechanisms of phenolic systems under different catalytic environments and elaborates on how precise regulation of process parameters enables the tailored design of aerogel microstructures.

### 2.1. Mechanisms of Competitive Reaction Kinetics Under Acid/Base Catalysis

The most established precursor system for phenolic aerogels is the polycondensation of phenol (or resorcinol, bisphenol A) with formaldehyde in a solvent. This process primarily comprises two stages: electrophilic addition (hydroxymethylation) and nucleophilic substitution (polycondensation bridging) [14,28]. The pH of the catalyst not only modifies the nature of the active sites but also dictates the colloidal particle morphology of the gel framework through regulating the relative rates (competitive kinetics) of these two steps [33].

**Figure 2:**
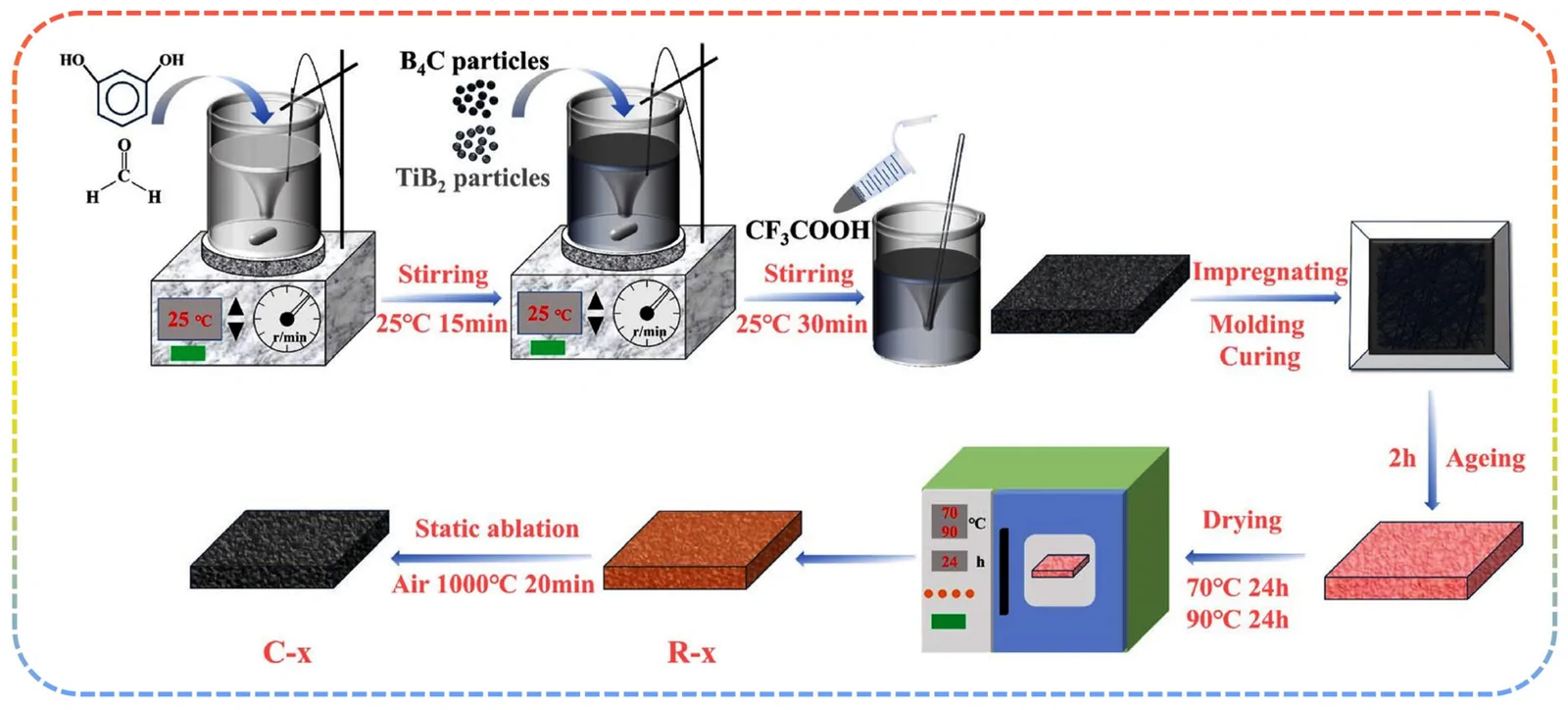
Preparation of TiB_2_/B_4_C Modified Phenolic Aerogel Composite [27].

Acid catalysis serves as a critical means for regulating the sol–gel process and final properties of phenolic aerogels. In acidic media, formaldehyde is protonated to generate a hydroxymethyl carbocation, which then undergoes electrophilic attack on the phenolic ring. Because the rate of hydroxymethyl condensation is much higher than that of the initial addition step, the reaction preferentially drives linear chain extension and physical entanglement rather than particle nucleation, ultimately yielding a uniform fibrous framework and dense micropores [33,34]. This mechanism strongly favors low-branched-chain propagation and triggers spinodal decomposition, which leads to skeleton coarsening and the formation of a hierarchical network dominated by macropores and broad mesopores. Although the specific surface area is reduced, shrinkage is significantly reduced, and rigidity is enhanced [34]. Based on this fundamental mechanism, researchers have achieved tailored microstructures and properties by regulating the type of acid and the reaction pathways. Zhao et al. reduced the curing activation energy from 300 kJ/mol to 90 kJ/mol, which accelerated protonation and gelation. Through the competition between rapid crosslinking and phase separation, they suppressed the non-uniform growth of macropores, resulting in a stable and uniform framework with a shrinkage of only 4.5% and a compressive strength of 5.7 MPa at 50% strain, thus achieving a combined enhancement of mechanical properties, thermal insulation, and fire resistance [35]. Gong et al. constructed an initial network under HCl catalysis. Then they leveraged the competitive reaction kinetics between carbonization and sulfonation—occurring during the induction of partial graphitization and the in situ introduction of sulfonic acid groups—to simultaneously enhance skeletal stability and mass-transfer capability, while also creating sulfonic acid active sites. This approach achieved a conversion rate of 95.3% in esterification with an activation energy of only 22.26 kJ/mol, while offering the advantages of low pressure drop and high flux [36]. Furthermore, Liu et al. extended the acid-catalysis principle to composite systems, employing the kinetic competition between phenolic polycondensation and filler sedimentation to suppress the agglomeration of TiB_2_/B_4_C and achieve uniform dispersion. In particular, the C–B_2_O_3_–TiO_2_–TiC multiphase barrier, generated by the competition between ceramisation and pyrolytic oxidation at high temperatures, plays a role in oxygen consumption and carbon fixation (Figure 2) [27].

**Figure 3:**
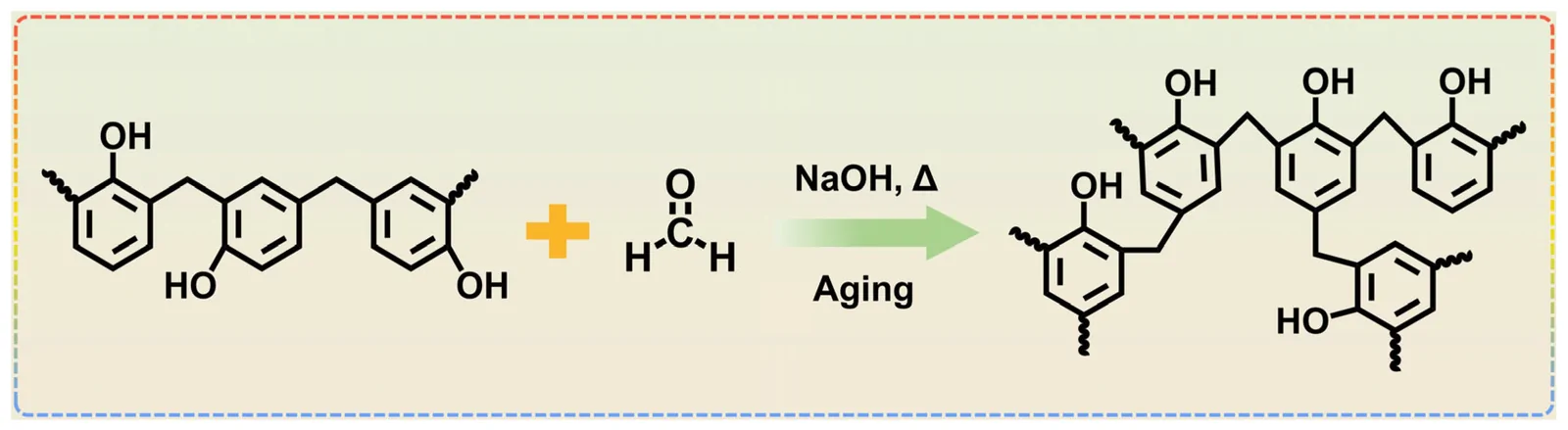
Preparation of phenolic aerogels under alkaline conditions [37].

Under alkaline conditions (Figure 3), the catalyst deprotonates the phenolic hydroxyl groups to generate phenoxide anions, which then undergo electrophilic addition with formaldehyde to introduce hydroxymethyl groups. Because the rate of hydroxymethylation is significantly higher than that of polycondensation, formaldehyde is rapidly consumed, forming highly branched oligomeric intermediates, which shortens the polycondensation process and increases the crosslinking density [37]. These branched intermediates nucleate in situ to form charged colloidal particles; electrostatic repulsion delays their aggregation, and eventually crosslinking locks the structure into a high-specific-surface-area microporous–mesoporous hierarchical network [34]. Based on this mechanism, Guo et al. found that in the competitive kinetics between sol–gel polymerisation and solvent evaporation driven by thermal decomposition of hexamethylenetetramine (HMTA) under base catalysis, a filtration-induced concentration gradient affects curing uniformity and that 4.7% HMTA achieves an optimal balance of density, thermal insulation, and mechanical properties [38]. Wu et al., utilising the competitive kinetics among base-catalysed phenolic polycondensation, solvent evaporation, and phase separation, constructed an interfacial evaporator with a gradient nanoparticle skeleton: the macroporous side features a hydrophobic photothermal layer for self-floating heat localisation, while the gradient capillary enhances water supply. Combined with ZnCl_2_/F127 templating to tailor the nanoparticle size from bottom to top, this design endows the aerogel with high light absorption (96.8%) and evaporation rate (1.64 kg/(m^2^·h)) [39].

In general, phenolic resins fall into two categories: thermosetting and thermoplastic. Thermosetting resins are formed via base-catalyzed hydroxymethylation of phenol with an excess of formaldehyde and subsequent polycondensation to yield prepolymers, which are then cured by heating or acid addition into an insoluble, infusible three-dimensional crosslinked network. Thermoplastic resins, in contrast, are typically prepared under acid catalysis with a low formaldehyde-to-phenol molar ratio, yielding prepolymers predominantly featuring linear or low-branched structures, and they generally require the addition of a curing agent such as hexamethylenetetramine (HMTA) to achieve further crosslinking [28]. The essential difference between the two lies in their dependence on curing agents and the pathways for constructing crosslinked networks.

### 2.2. Chemical Nature and Processing Characteristics of Resorcinol–Formaldehyde (RF) Aerogels

Resorcinol–formaldehyde (RF) aerogels and conventional phenolic (PF) aerogels both belong to the family of phenolic polycondensation materials, with RF systems being one of the common precursors for carbon aerogels. Compared with phenol, which contains only one phenolic hydroxyl group, resorcinol possesses two strongly electron-donating phenolic hydroxyl groups that can increase the electron density of the benzene ring through resonance effects, thereby rendering the ortho and para positions more nucleophilically active and favouring hydroxymethylation by formaldehyde as well as subsequent polycondensation. Consequently, under comparable catalytic conditions, RF systems generally exhibit higher reactivity and faster gelation kinetics. Meanwhile, the enhanced polarity conferred by the two phenolic hydroxyl groups in resorcinol improves its water solubility, facilitating uniform dissolution, reaction, and sol–gel transition of RF precursors in aqueous media. In contrast, phenol, being more hydrophobic, typically requires organic co-solvents, surfactants, or tailored catalytic systems to improve the dispersion and reaction homogeneity in its PF sol–gel process [5,14,40,41].

### 2.3. Strategies for Microstructure Regulation

Precise control over the microstructure of phenolic aerogels lies at the heart of tailoring their properties for specific applications. The sol–gel process is jointly governed by parameters such as the molar ratio of reactants to catalyst, solvent properties, and initial solid content, which collectively determine the nucleation density and growth kinetics of primary nanoclusters, thereby dictating the topological features of the gel framework [19,42].

The gelation of phenolic aerogels is governed by the interplay of organic polymerization kinetics, solvent effects, and polymerization-induced phase separation, giving rise to multiscale structural evolution [43]. Solvents dictate the timing of phase separation by modulating thermodynamic compatibility: water induces premature phase separation, resulting in macropores, whereas good solvents (e.g., ethanol/acetone) can delay phase separation, thereby locking in a uniform nanonetwork [44]. Adjusting solvent polarity and dielectric constant alters the thickness of the solvation layer, while high-boiling-point co-solvents (e.g., glycerol) can act as dynamic templates to preserve isotropic mesopores, offering flexible chemical routes for pore engineering [45].

**Figure 4:**
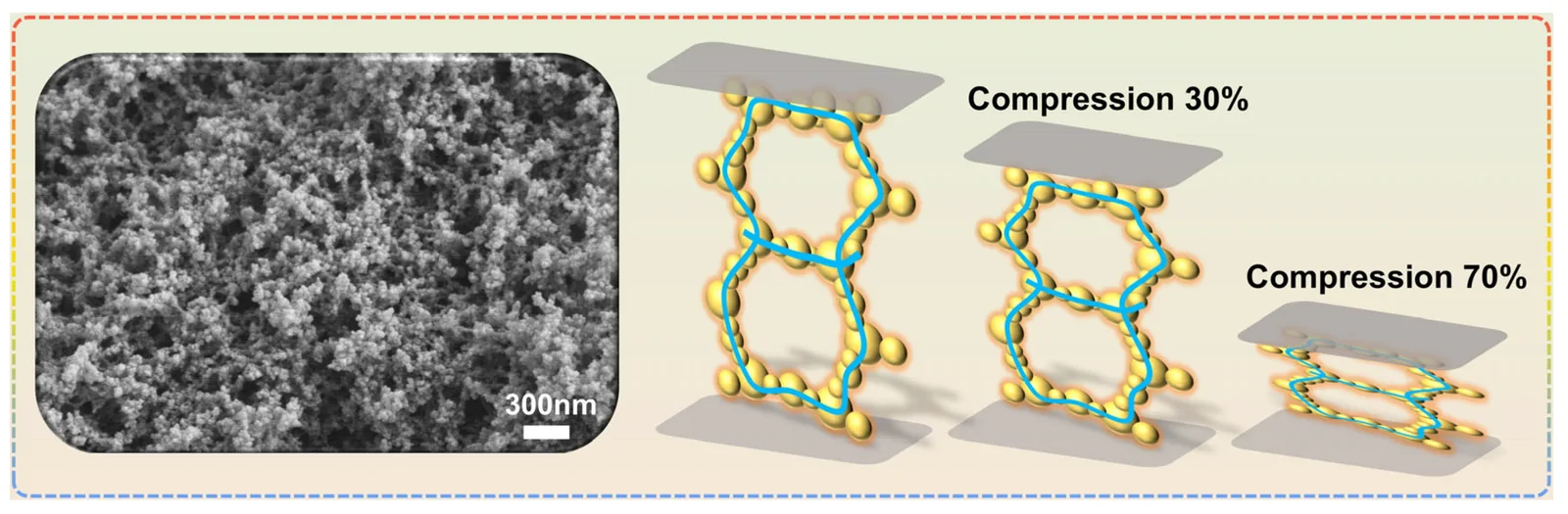
Microstructure and compressive-strengthening mechanism of phenolic aerogels [46].

The microstructure of phenolic aerogels plays a decisive role in balancing their mechanical and thermal insulation properties, and this structure can be effectively tailored by modulating the crosslinking density, phase-separation behavior, and component ratios during the sol–gel process (Figure 4) [46]. Fu et al. focused on the influence of the inorganic component ratio on phase separation and hybrid uniformity. They found that a high Si/Zr ratio induces microphase separation between the silicone and phenolic components, accompanied by skeleton coarsening. Reducing the Si/Zr ratio effectively suppresses phase separation, refines the particles to the nanoscale (45.9 nm, comparable to pure phenolic aerogels), and yields a co-continuous structure. At a Si/Zr ratio of 9:1, this system jointly constructs a dense C–SiO_2_–SiC–ZrO_2_ multiphase ceramic layer, resulting in a low linear ablation rate of 0.032 mm/s [47]. Meanwhile, Gu’s team systematically investigated the effect of HMTA concentration on the competition between crosslinking and phase separation. At a low concentration (1.2 wt%), a uniform grape-like porous network is formed; at 4.7 wt%, the mesoporosity becomes most abundant, and the thermal conductivity reaches its minimum, achieving a balanced trade-off between mechanical and thermal insulation properties. When the concentration is further increased to 11.2 wt%, excessive crosslinking triggers localised densification; pores disappear, and although mechanical properties are enhanced, thermal insulation performance deteriorates [38]. Building on this foundation, they further examined the regulatory effects of porogen content on the microstructural evolution of the aerogel composite system. At low porogen content, a dense network with fine particle size (0.735 μm) and a complete encapsulation layer is formed; at moderate content, a bicontinuous open-pore structure with grape-like clusters is constructed; at excessively high content, over-dilution leads to the formation of isolated large particles (1.518 μm) and loose aggregates, which disrupt the three-dimensional continuity and cause the compressive strength to drop from 1.92 MPa to 0.24 MPa [48]. We should also consider the potential impact of sample preparation and characterization methods on structural observations. The above studies indicate that precise control of both the phase-separation length scale and the network continuity is key to achieving a simultaneous improvement in the properties of phenolic aerogels.

## 3. Key Drying Processes and Interfacial Regulation Mechanisms

As is well known, upon completion of the sol–gel process, the three-dimensional nanoporous framework of the wet gel is pervaded by solvent molecules. Efficient removal of the solid/liquid media from these pore channels, while preserving the topological integrity of the nanonetwork, constitutes the central technological bottleneck in the fabrication of high-performance phenolic aerogels; the core challenge lies in preventing capillary-force-induced framework collapse and volumetric shrinkage. According to the Young–Laplace equation, the capillary pressure (ΔP, Pa) exerted on the pore walls is given by Equation (1) [49,50].

(1) $$\Delta P=\frac{2\gamma cos\theta }{r}$$

where *γ* represents the surface tension of the solvent (N/m), *θ* is the contact angle between the liquid and the pore wall (rad), and *r* is the pore radius (m).

Therefore, this chapter focuses on three mainstream drying techniques—supercritical drying, ambient-pressure drying, and freeze drying—and elucidates the interfacial regulation mechanisms and thermodynamic behavior that underlie their macroscopic processing routes.

### 3.1. Supercritical Drying (SCD)

**Figure 5:**
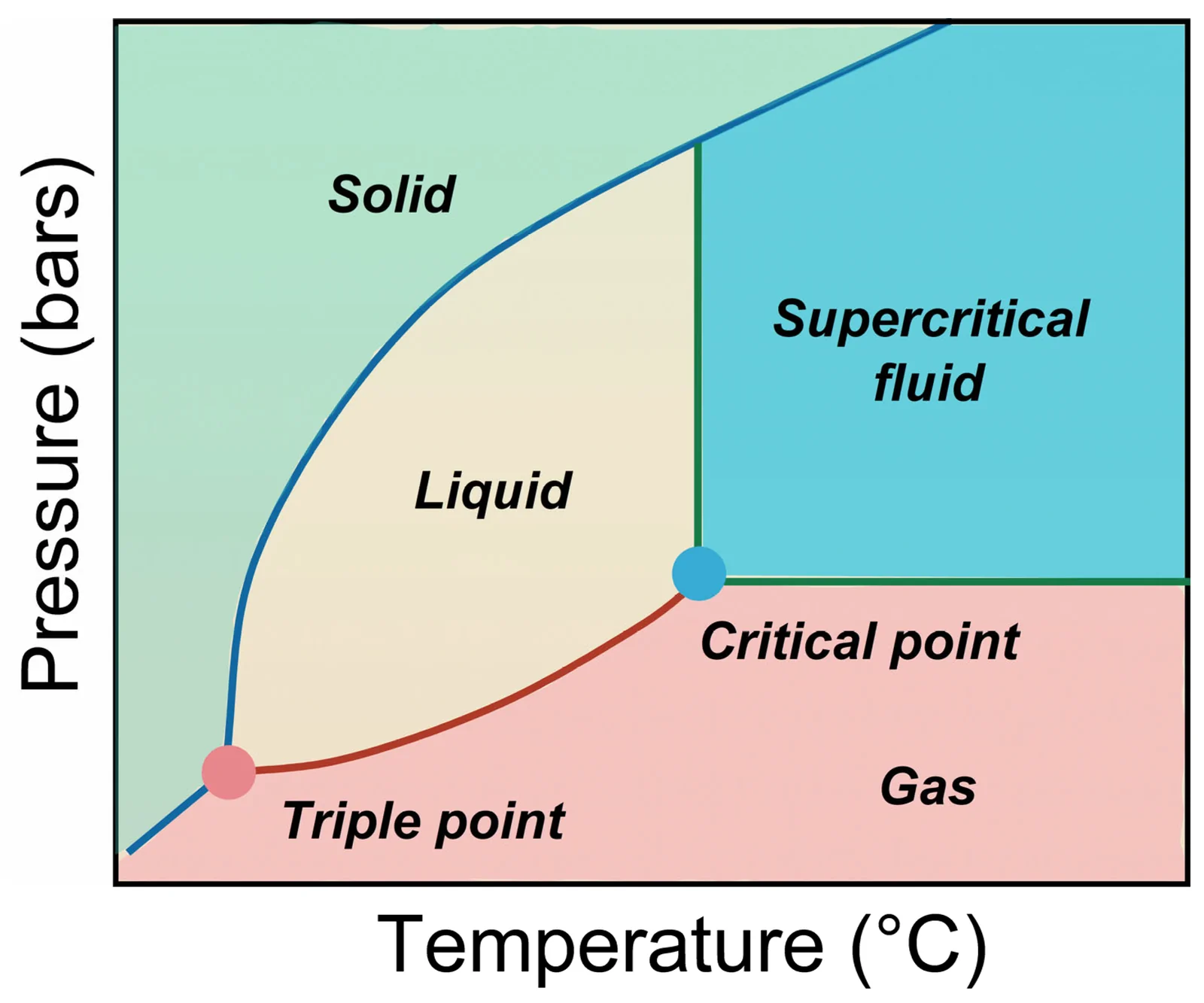
Phase equilibrium diagram for the solid, liquid, and gas phases.

SCD leverages the thermodynamic feature of vanishing gas–liquid interfacial tension under supercritical conditions to eliminate capillary pressure (Δ*P*) and is recognized as a classic technique for fabricating phenolic aerogels with high specific surface area and low density (Figure 5 and Table 1). CO_2_ supercritical drying removes the solvent under shear-stress-free conditions through liquid displacement under high pressure and isothermal depressurization venting, thereby maximizing the retention of the nanoscale framework’s structural integrity [16,51,52]. Building on this technique, Liu et al. observed that supercritical CO_2_ drying (45 °C, 8.3 MPa, 10 h) not only preserves the nanoporous framework of the phenolic gel intact and prevents capillary-force-induced pore collapse but also yields, after carbonization, a uniform boron-doped carbon structure in which the boron carbide phase enhances thermal resistance and disperses stress. This imparts the material with both a low thermal conductivity (0.0475 W/(m·K)) and a compressive strength of 1.94 MPa, improving high-temperature thermal insulation and structural stability [53]. Similarly, Li et al. demonstrated that supercritical CO_2_ drying (55 °C, 13 MPa) preserves the structural integrity of phenolic-based carbon aerogels through the coordinated shrinkage of fibers and matrix, which eliminates cracks. This yields a high specific surface area (438.92 m^2^/g) and abundant mesopores, achieving a low thermal conductivity of 0.027–0.164 W/(m·K) over the temperature range of 25–1800 °C, further validating the effectiveness of this technique in optimizing thermal insulation performance [54]. However, this technique is constrained by the molecular diffusion kinetics within the nanopores, leading to long displacement cycles, and the high-pressure operation imposes stringent demands on equipment safety and energy consumption, making it difficult to achieve continuous, large-scale production (Table 2). It is worth noting that CO_2_ is currently the predominant drying medium in phenolic systems. These high costs and harsh process conditions hinder its industrial scale-up; therefore, developing low-cost, continuous, and scalable drying processes while maintaining structural integrity is a critical direction for phenolic aerogel fabrication technology. However, it is by no means the optimal drying technique in a universal sense; its advantages lie primarily in applications that demand high added value, high performance, and stringent structural integrity.

### 3.2. Freeze-Drying (FD)

**Figure 6:**
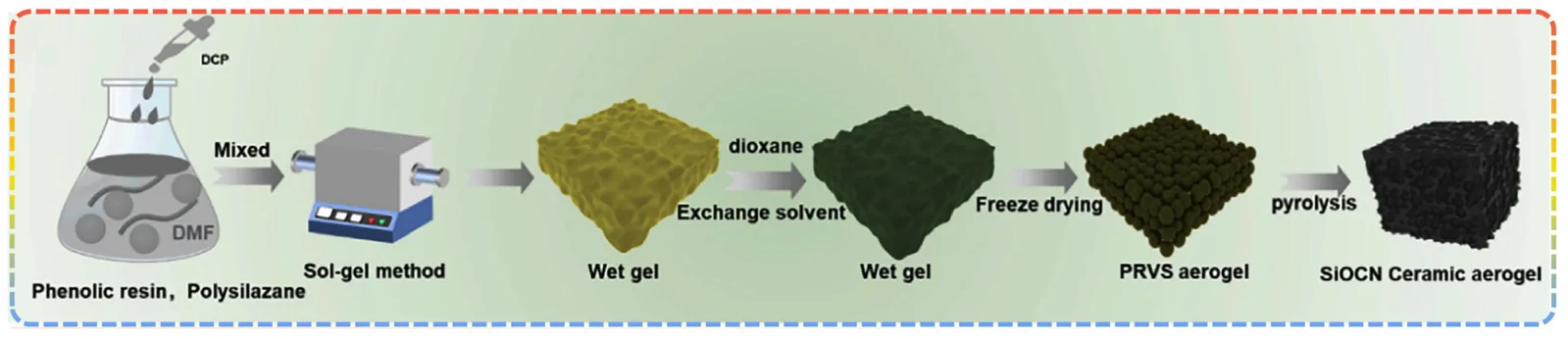
Schematic diagram of the ceramic aerogel preparation process [58].

FD transforms the gas–liquid interface into a gas–solid interface by rapidly freezing the wet gel and directly sublimating the solid solvent under vacuum, thereby effectively eliminating capillary stress. The core lies in modulating the nucleation and crystallization kinetics of the solvent and utilizing the ice-templating effect to directionally construct pore morphologies [16,56]. For instance, directional freezing techniques can drive crystal growth along a thermal gradient, endowing phenolic aerogels with anisotropic frameworks and achieving simultaneous optimization of axial load-bearing and radial thermal insulation. On this basis, researchers have successfully prepared a variety of functionalized aerogels. Li et al. preserved the phenolic nanofiber network (average diameter ~20 nm) intact via freeze-drying, and the resulting material exhibits both a high specific surface area and abundant pores. At 298.2 K and 1.0 bar, it shows an SO_2_ uptake capacity of 10.58 mmol/g and an SO_2_/N_2_ selectivity as high as 7271, demonstrating highly efficient and reversible capture capability [57]. Wang et al., by rapidly curing the three-dimensional framework to inhibit capillary shrinkage, obtained a hierarchical pore structure with a broad pore-size distribution, thereby endowing SiOCN ceramic aerogels with low density, low thermal conductivity, and enhanced microwave absorption properties, achieving a coupling of lightweight thermal insulation and electromagnetic absorption (Figure 6) [58]. However, the mechanical stress generated by solvent crystallization (e.g., volume expansion during water freezing) tends to disrupt the micrometer-scale gel network, leading to cracking of the material. To address this, replacing water with media that have a high freezing point and low solidification shrinkage—such as tert-butyl alcohol—can effectively inhibit crystal coarsening and preserve the nanoscale topological structure intact. He et al. employed tert-butyl alcohol exchange combined with freeze-drying to successfully retain the cobalt-coordination-enhanced resorcinol–formaldehyde gel network, which resembles a metal–phenolic network (MPN). The resulting aerogel features a thick-walled hierarchical pore structure with a density of only 0.09 g/cm^3^ and a thermal conductivity as low as 0.039 W/(m·K), and does not fracture even at a compressive strain of 68.6%, thereby achieving a simultaneous enhancement of mechanical and thermal insulation properties [33]. Overall, freeze-drying requires operation at low temperatures and under high vacuum, entailing considerable equipment investment and energy consumption. Moreover, its fine-tuning capability for pore structures and the overall integrity of the products remain inferior to those achieved by supercritical drying. Looking ahead, further breakthroughs are required in controlling ice-crystal growth, improving solvent-exchange efficiency, and ensuring the uniformity of large-sized samples.

### 3.3. Ambient-Pressure Drying (APD)

**Figure 7:**
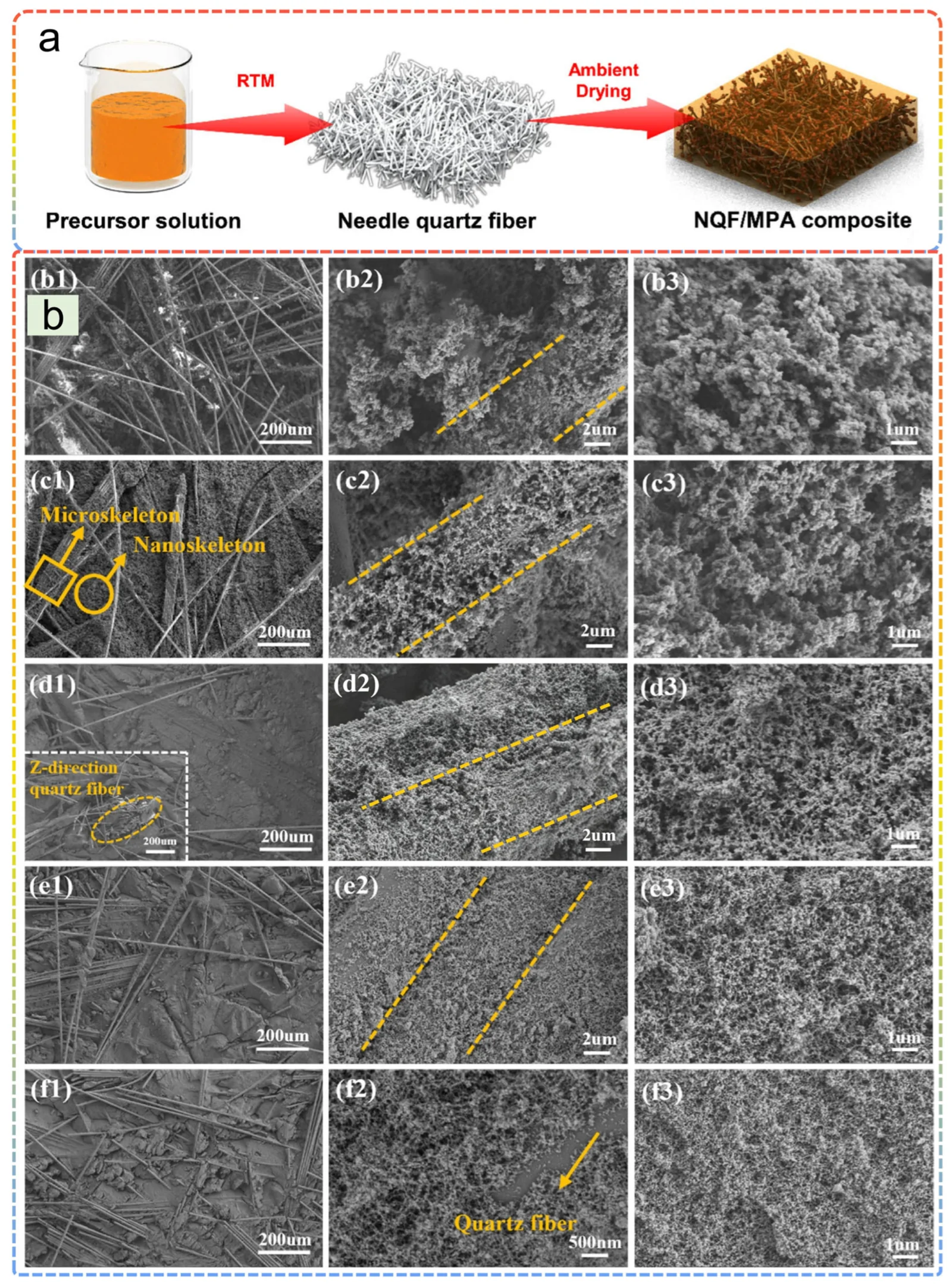
(**a**) Flowchart for the preparation of multifunctional phenolic composite aerogels via APD [60]; (**b**) microstructure of NQF/PR aerogel composites, where (**b1**–**b3**) NQF/PR-10; (**c1**–**c3**) NQF/PR-15; (**d1**–**d3**) NQF/PR-20; (**e1**–**e3**) NQF/PR-25; (**f1**–**f3**) NQF/PR-30 [61].

APD offers a viable industrial route to overcome the reliance on high-pressure equipment in supercritical drying and the uncontrollability of ice-crystal growth in freeze-drying. Its core lies in suppressing framework shrinkage and structural collapse by modulating the capillary stress during solvent evaporation. Mainstream strategies include: (i) solvent exchange with low-surface-tension solvents (e.g., n-hexane) to directly reduce capillary stress; (ii) silane functionalization lowers the surface energy of pore walls and modifies their wettability by grafting hydrophobic groups, thereby substantially reducing the tensile stress exerted by capillary forces on the skeleton during drying, while also promoting the reversible recovery of drying shrinkage and the restoration of structural elasticity; and (iii) construction of hybrid interpenetrating networks to enhance the shrinkage resistance and stiffness of the framework [16,22]. Building on these strategies, Lu et al. employed phosphorylated hollow microspheres to inhibit drying shrinkage, yielding a phenolic composite aerogel with a linear shrinkage of only 4.32% and a density as low as 0.244 g/cm^3^. The retained mesoporous–macroporous structure (specific surface area: 66.3 m^2^/g), combined with a micro–nano dual-sphere configuration, achieves a synergy of low thermal conductivity (0.045 W/(m·K)) and high compressive strength (4.16 MPa) [59]. To address the brittleness issue of phenolic aerogels, we found that a porous structure with a solid content of 20% possesses a pearl-necklace-like bimodal mesoporous network that combines high specific surface area, low thermal conductivity, and dimensional stability, achieving an optimal balance between porosity and structural rigidity [25]. Long’s group, through melamine copolymerization to reinforce the crosslinked network, successfully preserved the uniform mesoporous structure, verifying the effectiveness of chemical crosslinking enhancement for structural stability (Figure 7a) [60]. Zhao et al. further tuned the precursor concentration, refining the phenolic skeleton to 38.91 nm and reducing the pore size to 49.53 nm, forming a compact nanoporous structure, and combined it with fiber reinforcement to suppress gaseous heat transfer (with solid-phase heat transfer contributing over 80%), reducing thermal conductivity to 0.0646 W/(m·K) while increasing compressive strength to 7.70 MPa (Figure 7b) [61].

**Table 2:** Comparison of different drying methods [62,63].

Drying Technology	Equipment Requirements	Production Cost	Advantages	Drawback
SCD	Pressure vessel	High energy consumption, long cycle, high temperature, and high pressure	Low shrinkage, high porosity, stable performance	Complex equipment, large investment, high cost, limited scale
FD	Vacuum freezing system	High energy consumption, long cycle, low temperature, and high vacuum	Low shrinkage, large surface area, dries thoroughly, and is eco-friendly	Poor structural integrity, difficult to scale up
APD	Conventional drying equipment	Low energy consumption, normal pressure	Easy to operate, suitable for large-scale production	Significant shrinkage, structural vulnerability, insufficient strength

Notably, the thermal conductivity values reported in the literature exhibit considerable discrepancies, which cannot be attributed solely to the drying method but are also closely related to factors including precursor concentration, catalyst system, pore size distribution, density, sample moisture content, and testing conditions.

It is worth noting that, in addition to the solvent- and surface-modification routes described above, alternative techniques such as microwave drying and vacuum drying have also been explored for the preparation of high-performance aerogels. For instance, Li et al. found that vacuum drying effectively prevents capillary shrinkage and preserves the high compressive strength. The resulting coal-tar-based phenolic carbon aerogel exhibits superhydrophilicity, low thermal conductivity, and excellent compressive strength, achieving an evaporation rate of 2.23 kg/(m^2^·h) and an energy efficiency of 92.5% [64]. However, as shown in Table 2, each drying technique has its own advantages and limitations; therefore, the optimal choice should be made on a case-by-case basis according to the specific requirements. Overall, APD offers significant advantages in equipment cost and operational convenience; however, its ability to precisely preserve microporous structures still falls short of that achieved by SCD. Future efforts should focus on continuous optimization of solvent-exchange efficiency, enhancement of framework rigidity, and uniformity of large-sized samples (Table 2).

### 3.4. Carbonization Process

Phenolic resin aerogels, owing to their high char yield, serve as precursors for the preparation of carbon aerogels. The carbonization process transforms the organic framework into an amorphous carbon skeleton under an inert atmosphere, imparting to the material electrical conductivity, enhanced thermal stability, and an optimized pore structure [65]. Carbonization temperature and heating rate are key parameters for tuning the final properties: pyrolysis at lower temperatures (around 600 °C) is insufficient, leaving residual organic composite structures, whereas higher temperatures (≥900 °C) enhance graphitization and electrical conductivity, although care must be taken to avoid excessive shrinkage or even collapse of the pore structure. For example, Huang et al. reported phenolic resin carbon aerogels prepared via a tube furnace with a hierarchical macro-/microporous structure, which exhibited high mechanical strength and low thermal conductivity [66]. However, it should be noted that the trade-off among electrical conductivity, specific surface area, and structural integrity depends on the carbonization conditions.

Co-carbonization involves the simultaneous thermal treatment of phenolic aerogels and fibrous reinforcements, allowing the matrix and reinforcements to shrink together and thus reducing interfacial thermal mismatch stresses. This approach enables the fabrication of large-sized, crack-free composites, which are suitable for high-temperature insulation and aerospace thermal protection. In addition, the introduction of heteroatoms such as boron during carbonization for in situ modification can generate secondary phases like boron carbide, which utilize multiple thermal reflections at phase interfaces to enhance thermal insulation and improve mechanical performance. For example, Gao et al. explored the thermal stability mechanism of boron-modified phenolic resin carbon aerogels [67]. This integrated strategy—combining carbonization with in situ modification—offers a significant technological pathway for the development of lightweight, ultrahigh-temperature-resistant, and multifunctional carbon aerogel composites.

## 4. Properties and Structure–Property Relationships

Although sol–gel processing and drying technologies have enhanced the nanoporous structural integrity of phenolic aerogels, the fragile interparticle neck connections still give rise to poor mechanical properties and limited oxidation resistance, which severely constrain their reliability under high-temperature service conditions. Their macroscopic properties are fundamentally governed by the cross-scale structure–property relationships that span from molecular structure and network topology to pore architecture and ultimately to macroscopic performance. Therefore, achieving simultaneous optimization of structure and performance through framework reinforcement, network modulation, and high-temperature-resistant functionalization is the key to expanding the applications of phenolic aerogels in extreme thermal protection, electromagnetic shielding, and other related fields.

### 4.1. Mechanisms of Thermal Conductivity Regulation by Structure

**Figure 8:**
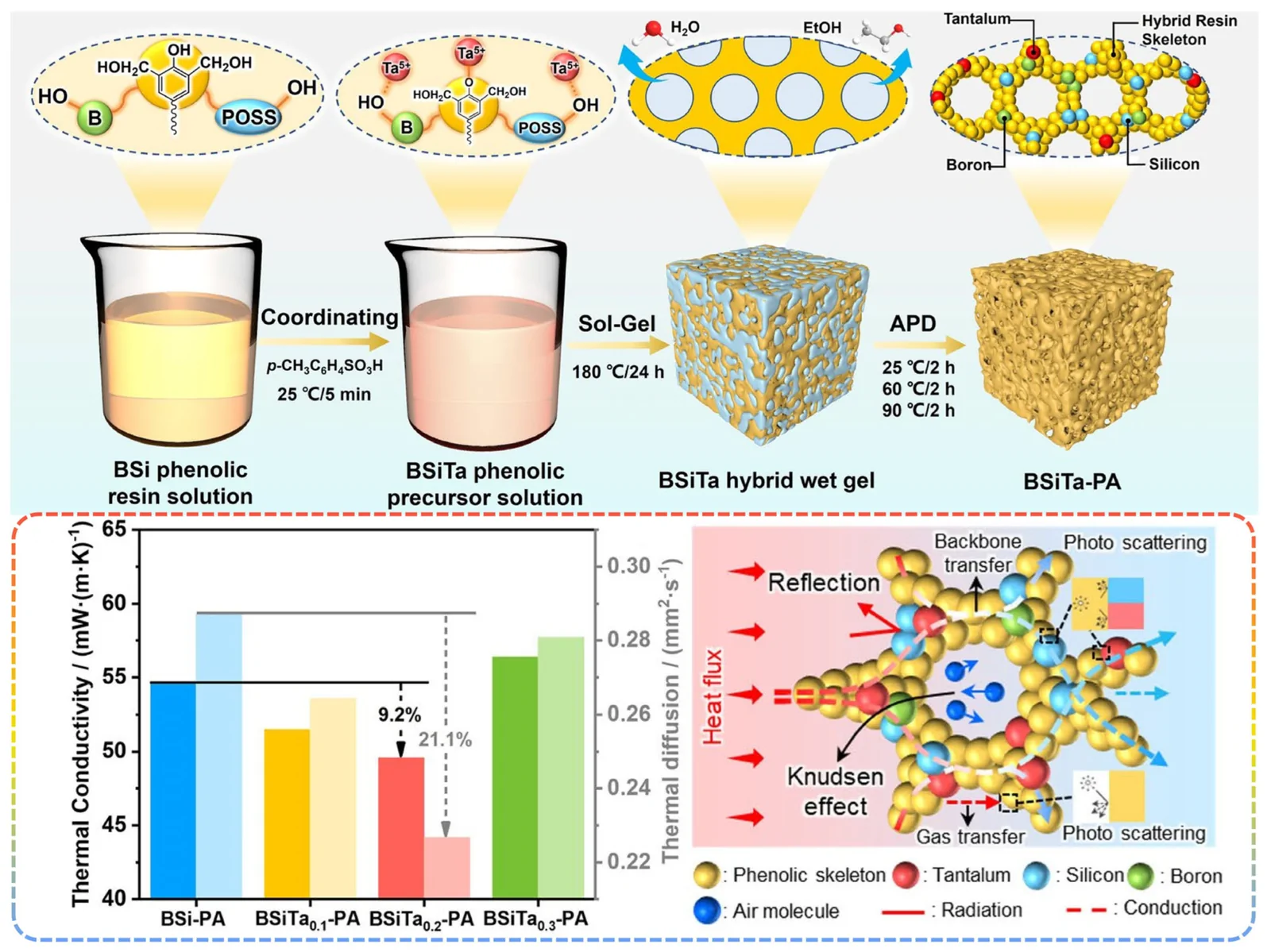
Schematic illustration of the preparation process and thermal conductivity/heat transfer mechanisms of multifunctional aerogels [70].

The thermal transport in phenolic aerogels is governed by four concurrent contributions: solid conduction (*λ*_s_), gaseous conduction (*λ*_g_), radiative transfer (*λ*_r_), and convective heat transfer (*λ*_con_) (Equation (2)). The pore architecture exerts a decisive influence on the overall thermal conductivity [24,68]. When the pore size approaches or falls below the mean free path of gas molecules, the Knudsen effect becomes prominent, shifting the dominant collision mechanism from intermolecular collisions to molecule–wall collisions, thereby effectively suppressing gaseous thermal conduction (Equations (3)–(5)) [68]. Concurrently, the discontinuous skeleton arising from high porosity and low density elongates the heat-flow pathways, thereby diminishing the contribution of solid conduction. Moreover, the highly crosslinked aromatic network can evolve into a stable carbonaceous framework during pyrolysis, further enhancing structural integrity at elevated temperatures and counteracting the increase in radiative transfer. Therefore, the design core for achieving ultra-low thermal conductivity insulating materials lies in the synergistic regulation of crosslinking density, pore-size scale, and pore-structure uniformity to simultaneously suppress solid, gaseous, and radiative heat transfer. Within this framework, Ning et al. utilized a silicone prepolymer (KH560-MTMS) to chemically bond with the phenolic network via epoxy groups, thereby constructing a thick-walled nanostructure. Although this structure temporarily increased solid thermal conduction during the early stage of densification, the subsequently formed hierarchical meso/macroporous structure effectively reinforced the Knudsen effect and, together with the phonon-scattering barrier provided by Si-O bonds, stabilized the thermal conductivity at 0.0450 W/(m·K) [69]. Distinct from the aforementioned phonon-suppression strategy dominated by siloxane bonds, Niu and co-workers introduced POSS cage-like structures into a boron–silicon–tantalum ternary hybrid system. The multiscale micro- and nanopores suppressed gaseous heat flow, while the Ta-O/Si-O covalent bonds created a strong phonon-scattering barrier, substantially reducing solid conduction through the framework. The synergy between these two effects further lowered the thermal conductivity to 49.6 mW/(m·K) (Figure 8) [70]. These findings indicate that, whether through mesoscale pore-structure optimization or atomic-level bonding regulation, the synergistic suppression of solid and gaseous heat transfer by multiple mechanisms constitutes a common pathway for enhancing the thermal insulation performance of phenolic aerogels.

(2) $$\lambda_{eff}=\lambda_{g}+\lambda_{s}+\lambda_{r}+\lambda_{con}$$

(3) $$\lambda_{g}=\frac{1}{1+2K_{n}\beta }\lambda_{g}^{0}$$

(4) $$K_{n}=\frac{\Lambda_{g}}{\delta }$$

(5) $$\Lambda_{g}=\frac{k_{B}T}{\sqrt{2}\pi d_{g}^{2}p_{g}}$$

where *K*_n_ is the Knudsen number, *β* is the energy transfer efficiency, $\lambda_{g}^{0}$ denotes the free gas thermal conductivity, and *δ* is the pore diameter. Λ*_g_* is the mean free path, *k*_B_ is the Boltzmann constant, *T* is the temperature, *d*_g_ is the average diameter of air molecules, and *p*_g_ is the gas pressure.

### 4.2. Mechanical Toughening Mechanisms

The optimization of the mechanical properties of phenolic aerogels centers on establishing a multiscale energy-dissipation mechanism that enables the simultaneous regulation of stiffness and toughness. At present, molecular-chain flexible modification has emerged as one of the most effective toughening strategies, primarily achieved through the introduction of flexible segments via chemical bonding or through the regulation of nanophase separation [71]. Ye et al. utilized an isocyanate coupling agent to achieve molecular-level chemical bonding between phenolic and silicone components, constructing a stable three-dimensional network that strengthens interparticle neck connections and incorporates flexible segments. The resulting material achieved a compression rebound rate of 70% while maintaining a low density (0.118 g/cm^3^) and low thermal conductivity (0.0318 W/(m·K), suppressing brittle fracture [72]. Yang et al., through controlled nanoscale phase separation, prepared PU-modified phenolic aerogels featuring a sub-10 nm co-continuous phase structure. The PU flexible segments dissipate compressive energy and alleviate stress concentration at the neck regions, endowing the material with a toughening effect: a compressive strength of 29 MPa and a modulus of 112 MPa at 60% strain, together with a recovery rate of 90% after 50% compression [73]. In addition, the introduction of flexible siloxane segments through prepolymer covalent bonding can refine the gel particle size and reconstruct the three-dimensional network, thereby promoting effective stress transfer and dissipation and simultaneously enhancing both toughness and strength [69]. Therefore, optimizing the crosslinked network and stress distribution through flexible-segment strategies can effectively improve the brittle failure mode of phenolic aerogels.

### 4.3. Mechanisms of High-Temperature Resistance and Anti-Oxidation Modification

Inorganic hybridization serves as a core strategy for enhancing the high-temperature resistance of phenolic aerogels, introducing silicon, boron, and transition metals into the framework via in situ polymerization, powder doping, or post-modification to construct an organic–inorganic synergistic network [74,75,76]. Among them, silicon species can form stable Si–O networks or in situ ceramic phases, improving thermal stability and oxidation resistance. Boron species, on the other hand, leverage their high-bond-energy crosslinked structures and the high-temperature formation of protective phases such as B_2_O_3_ and B_4_C to effectively delay oxidative ablation and enhance thermal insulation. Long et al. introduced nitrogen doping via melamine copolymerization, which generates more gaseous products during pyrolysis to enhance the gas-blocking effect. This works in synergy with the porous carbon phase, reducing the equivalent thermal conductivity at 1000 °C to 0.045 W/(m·K), and during arc-jet wind-tunnel testing at 130 Kw/m^2^ for 1000 s, the linear ablation rate was as low as 2.51 × 10^−6^ m/s [60]. Fu et al., by contrast, focused on zirconium-based hybridization. Nanoscale Y_2_O_3_ doping creates an inlaid structure that rapidly dissolves with ZrO_2_ at high temperatures to form the dense yttria-stabilized zirconia (YSZ), which eliminates phase-transformation stress and creates a dense thermal barrier layer. Concurrently, it catalyzes the graphitization of pyrolytic carbon into multilayered graphene, leading to a 33% reduction in the linear ablation rate under oxyacetylene flame conditions (2.0 MW/m^2^, 60 s) [77]. The introduction of OH-MWCNTs further promotes the graphitization of pyrolytic carbon and induces the in situ formation of a ZrO_2_/SiC multiphase ceramic layer during ablation. Coupled with the hierarchical pore structure that suppresses thermal conduction, this achieves an ultra-low linear ablation rate of 0.034 mm/s under oxyacetylene flame conditions (2.0 MW/m^2^, 60 s) [78]. These findings collectively indicate that the synergistic construction of multiscale phase transformations and a dense protective layer not only effectively resist heat flow erosion but also enhances the structural integrity of the material, providing a clear direction for the design of high-performance ablation-resistant phenolic aerogels.

## 5. Applications

Benefiting from precise sol–gel regulation and multifunctional modification, phenolic aerogels and their derived carbon counterparts have achieved a synergistic enhancement of mechanical and high-temperature resistance properties. Leveraging their advantages of ultralow thermal conductivity, high specific surface area, and high char yield, these materials exhibit great promise for applications in thermal protection, environmental remediation, and advanced energy storage.

### 5.1. Aerospace Thermal Protection and Thermal Insulation for Extreme Environments

**Figure 9:**
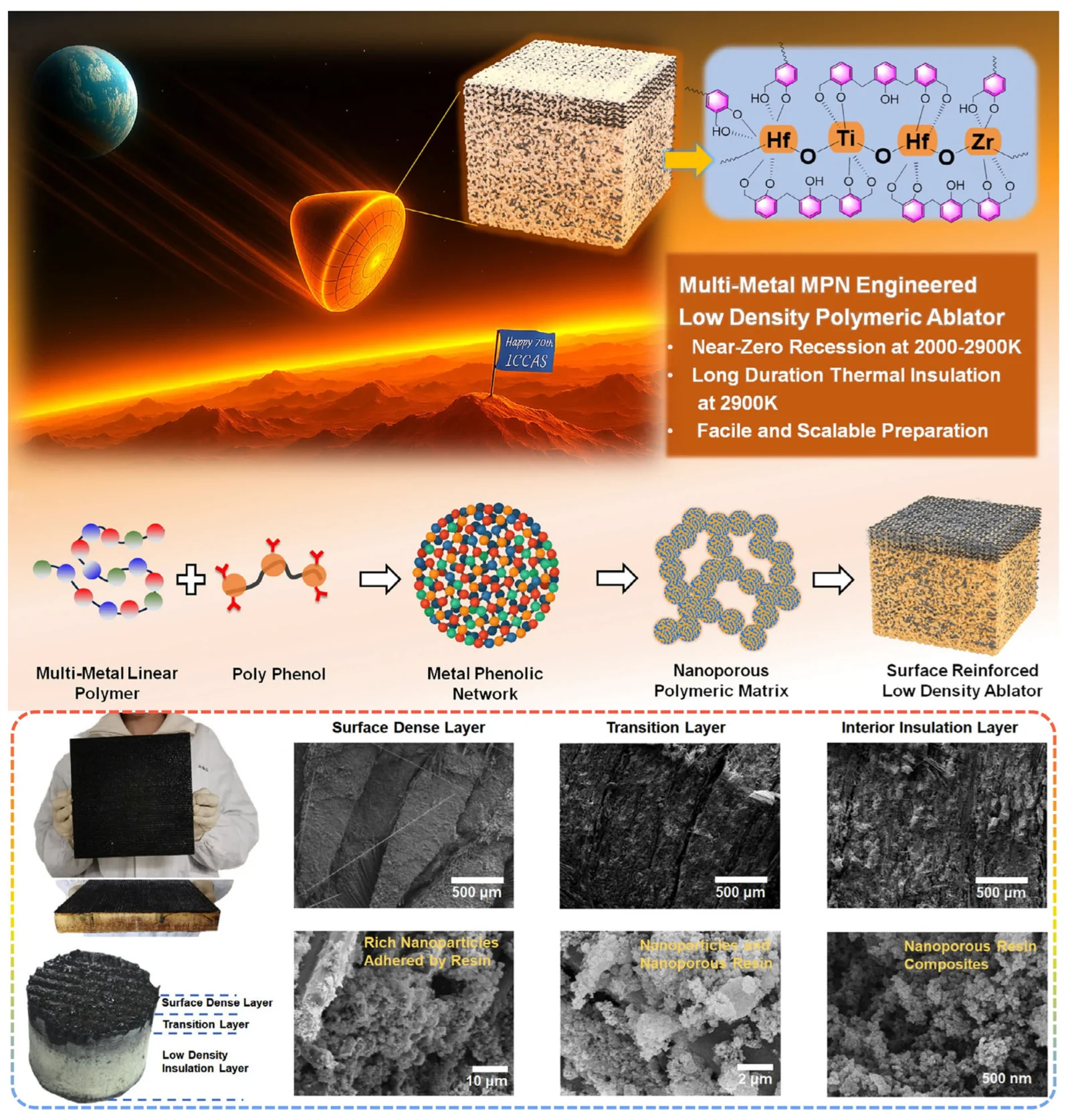
Design and post-ablation morphology of metal–phenolic network-based ablative materials [85].

Phenolic aerogels, with their ultralow thermal conductivity, high char yield, and excellent thermal stability, have emerged as a leading candidate material for aerospace thermal protection systems. Their micro-/nanoscale and three-dimensional continuous structure synergistically suppresses both solid and gaseous heat transfer, while high-temperature pyrolysis yields a stable carbonaceous framework. Building on this, the incorporation of fiber reinforcement and heterogeneous components—such as silicon, boron, and ultra-high-temperature ceramics—can further realize multifunctional simultaneous protection against ablation and oxidation [23,79,80,81]. To address the trade-off between lightweight design and ultra-high-temperature tolerance under extreme heat fluxes, researchers have developed various simultaneous modification strategies at both the interface and matrix levels. For instance, to counter the thermo-oxidative failure of phenolic aerogels, we introduced Ti_3_SiC_2_ and LaB_6_ as functional fillers to fabricate a fiber-reinforced phenolic aerogel composite that combines lightweight, high strength, and low thermal conductivity and that can in situ form a B–C–O–Si–Ti–La multiphase ceramic layer at high temperatures. The synergy of oxygen consumption, self-healing, and barrier mechanisms reduces the ablation rate and achieves long-lasting thermal insulation [24,26]. Ding et al. developed a silicon- and boron-modified phenolic aerogel composite that exhibits a thermal decomposition temperature up to 603.2 °C and a linear ablation rate of 0.077 mm/s under oxyacetylene flame ablation (2.0 MW/m^2^, 30 s). The dense C–SiO_2_–B_2_O_3_ multiphase ceramic layer formed in situ on the ablated surface effectively reflects the incident heat flux and suppresses matrix pyrolysis, thereby markedly improving resistance to thermal erosion [82]. Fu et al. synthesized a co-continuous silicon–zirconium hybrid phenolic resin via a two-step method, which was subsequently converted into a quartz-fiber-reinforced phenolic aerogel through a sol–gel process. This reduced the linear ablation rate to 0.032 mm/s (2.0 MW/m^2^, 60 s) while maintaining the back-face temperature below 200 °C [47]. Ye et al. further constructed a Si–Zr binary ceramic coating on the surface of carbon fibers, yielding a lightweight phenolic aerogel composite with a density of approximately 0.70 g/cm^3^. The dense SiO_2_–ZrO_2_ layer, through phase-change endothermic effects and viscous flow, achieves multiple functions, including oxygen barrier, thermal insulation, and crack sealing. The composite retains 47.5 wt% of its mass at 1000 °C and exhibits a low linear ablation rate of 0.0160 mm/s (2.0 MW/m^2^, 60 s), establishing a new paradigm for integrated interface–matrix design in extreme thermal protection [83]. Although the aforementioned silicon-, boron-, and zirconium-modified systems each offer distinct advantages, the densification and long-term stability of their ceramic layers remain critically challenged under ultra-high-temperature (>2500 K) and prolonged service conditions. Metal–phenolic networks (MPNs), as an important class of coordination network materials, have attracted widespread attention [84]. Yang et al. fabricated a multimetallic phenolic aerogel composite that exhibits a linear ablation rate of only 0.0017 mm/s at 2800 K and 0.0031 mm/s at 2900 K, while maintaining a back-face temperature rise of only 369 K after thermal testing at 2500 K for 1500 s. This outstanding performance is attributed to the in situ ceramization of the multimetallic components at high temperatures, which generates a dense interpenetrating oxide layer and an internal mass-fractal carbon–ceramic network. The synergy between these two structures enables near-zero material recession and long-lasting thermal insulation, fundamentally circumventing the traditional trade-off between lightweight design and ultra-high-temperature tolerance (Figure 9) [85]. Collectively, phenolic aerogel thermal protection materials are evolving along a clear trajectory—from matrix doping/modification through fiber interface coating to multimetallic in situ ceramization—with their temperature tolerance having expanded from conventional heat fluxes (<2000 K) to ultra-high-temperature extreme environments (>2800 K).

Owing to their intrinsically high char yield, high-temperature resistance, and low thermal conductivity, phenolic aerogels have positioned extreme thermal protection as the application domain that is currently closest to large-scale industrial implementation.

### 5.2. Adsorption, Separation, and Environmental Functional Materials

Phenolic aerogels and their carbonized derivatives, featuring three-dimensional nanoporous channels, not only provide efficient pathways for mass transport but also endow the materials with abundant active sites. Through pore-size engineering and surface functionalization—such as the introduction of amino groups, silane moieties, or heteroatom doping—these materials enable the selective and high-capacity capture of heavy metal ions, organic dyes, and CO_2_ [57,86]. Concurrently, hydrophobic modification can markedly enhance oil–water separation efficiency, whereas carbonization or activation treatments further optimize the microporous structure and adsorption kinetic performance. This synergistic design—integrating pore architecture and surface chemistry—endows the materials with a combination of excellent adsorption selectivity, high capacity, and good recyclability in water remediation and industrial gas treatment [87]. Gong et al. adopted a strategy of simultaneous sulfonation and carbonization using concentrated sulfuric acid, which introduced R-SO_3_H strong acid groups while preserving the three-dimensional interconnected framework. The resulting phenolic aerogel exhibited maximum adsorption capacities of 500.4, 299.8, and 339.9 mg/g for Pb^2+^, Cu^2+^, and Cd^2+^, respectively. The adsorption mechanism is primarily governed by ion exchange and chelation coordination, as further confirmed by DFT calculations, which identified Pb^2+^ as having the most favorable binding affinity. The material also demonstrated good recyclability and potential for scalable fabrication [87]. In a complementary study, Zhang et al. used acetic acid as an additive in combination with KOH activation to prepare a hierarchically porous phenolic-based carbon aerogel (specific surface area: 1205 m^2^/g, pore volume: >1.60 cm^3^/g). This material exhibited an adsorption capacity for methylene blue up to 1.25 times its own mass, with an equilibrium time of only 240 min. The adsorption behavior followed the pseudo-second-order kinetic model and the Langmuir isotherm, providing a viable strategy for the development of high-efficiency adsorbents for organic pollutants under ambient-pressure drying routes [86].

### 5.3. Electromagnetic Interference Shielding and Functional Composites

**Figure 10:**
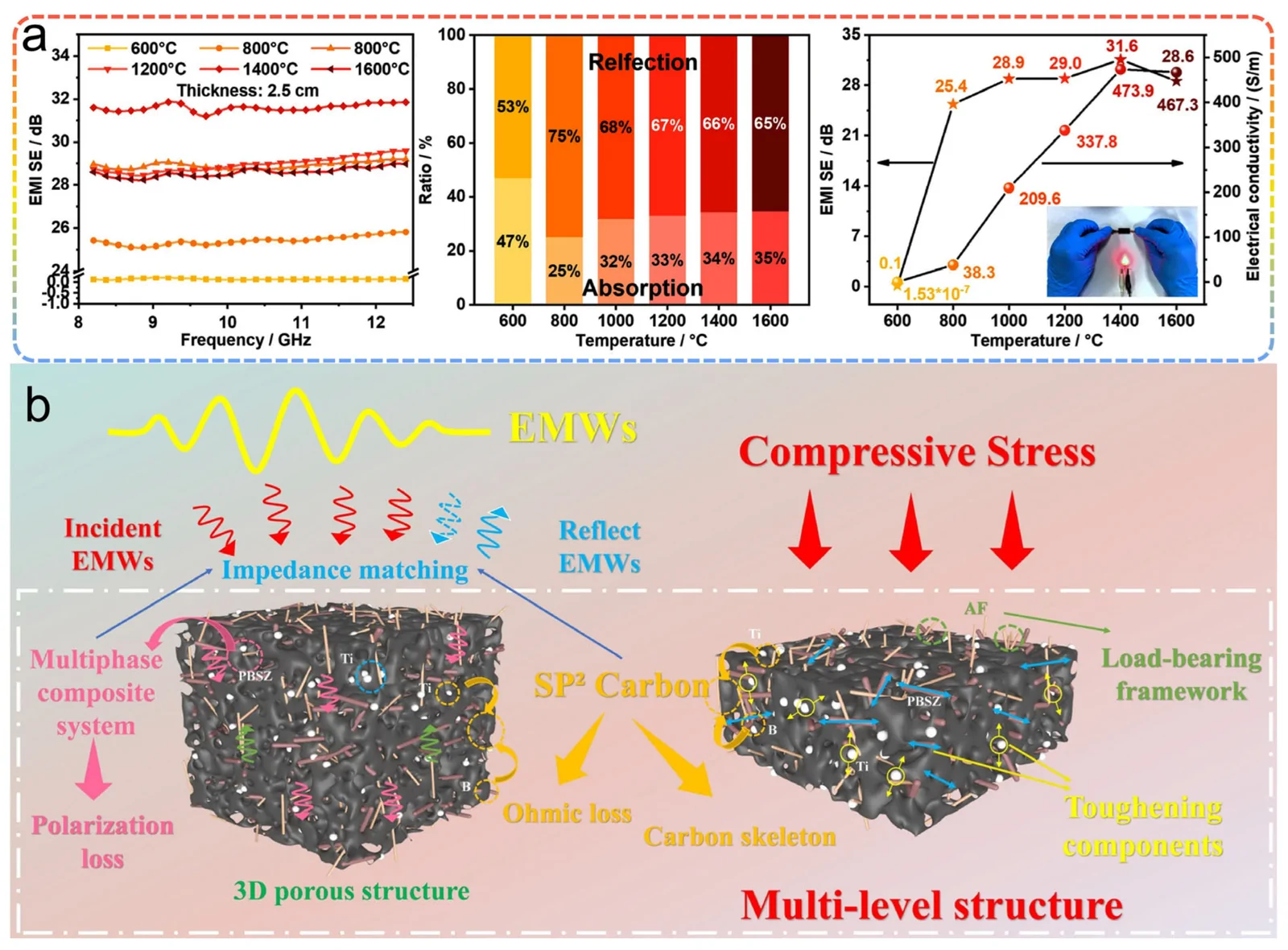
(**a**) EMI Shielding Performance and Electrical Conductivity [70]; (**b**) schematic illustration of the synergistic mechanism between mechanical properties and EMI shielding performance of ternary phenolic carbon aerogel composites [93].

Phenolic aerogels, with their three-dimensional porous framework and tunable conductive networks (e.g., graphene, carbon nanotubes, MXene), exhibit distinctive advantages in electromagnetic interference (EMI) shielding and absorption. Their hierarchical pore architecture not only endows the materials with lightweight characteristics but also enhances electromagnetic wave energy dissipation through conductive loss, interfacial polarization loss, and internal multiple scattering. Concurrently, the intrinsic impedance mismatch and confinement effects effectively prolong the propagation pathways of electromagnetic waves, further boosting attenuation efficiency [88]. Building on this foundation, the introduction of magnetic components or in situ carbonization strategies can further optimize impedance matching, transitioning the materials from a reflection-dominant behavior toward a low-reflection, high-absorption mode. This thereby satisfies the comprehensive requirements of high-performance electromagnetic protection—namely, “thin, lightweight, broadband, and strong” (Figure 10a) [70,89,90]. Following this design philosophy, research efforts in recent years have progressively shifted from the construction of single-component conductive frameworks toward multiphase synergies, interface engineering, and multifunctional integrated systems. For instance, Zhao et al. grew SnO_2_ nanorods in situ within a phenolic-derived carbon framework, constructing abundant heterointerfaces that introduce interfacial polarization relaxation. The resulting CA/SnO_2_ NRs composite exhibited a density of only 0.33 g/cm^3^ yet achieved an EMI shielding effectiveness of 36.6 dB (with an absorption contribution of 32.1 dB), realizing lightweight and efficient shielding [65]. Wang et al., by contrast, adopted a polymer-derived ceramic route to prepare SiOCN ceramic aerogels (density: 0.15–0.27 g/cm^3^; thermal conductivity: 0.03–0.04 W/(m·K)) with a macro-/mesoporous structure via freeze-drying. At a thickness of 1.2 mm, these materials exhibited a reflection loss of −31.6 dB at 16.48 GHz and an effective absorption bandwidth of 3.85 GHz, achieving synergistic thermal insulation and broadband strong absorption [58]. Furthermore, Liu et al. utilized N → B coordination to stabilize heterointerfaces, yielding a homogeneous organosilicon/boron-modified phenolic aerogel with domain sizes below 20 nm (specific strength: 1400 N·m/kg; mass retention > 50 wt% at 1000 °C). Upon pyrolysis, the material developed a gradient porous structure. Notably, the EMI shielding effectiveness of the sample containing 50% SiR increased rather than decreased (by 2.5%), whereas that of pure phenolic aerogel dropped by 73%, highlighting the adaptive shielding characteristics of the hybrid system under extreme thermal environments [91]. Building on these works, multifunctional integrated design has emerged as a new frontier. Wang et al., through molecular engineering, prepared a hydroxyl-functionalized CaCu_3_Ti_4_O_12_(CCTO-OH)/boron-phenolic/carbon-fiber aerogel that not only integrates thermal stability at 1300 °C, flame retardancy (with heat release and smoke release reduced by 54% and 44%, respectively), EMI shielding (28 dB), and piezoresistive sensing capabilities, but also incorporates a Bayesian-optimized CNN–LSTM model for thermal runaway early warning (with 99.99% accuracy) and employs a triboelectric nanogenerator(TENG) module for fall detection (98.7% accuracy) [92]. Guan et al., by contrast, constructed a triple-network titanium–boron–silicon phenolic aerogel at the molecular level through covalent coordination (density: 0.204 g/cm^3^; elastic recovery >92% after 50% compression; compressive strength: 1.97 MPa). This material withstands ablation at 1300 °C for 30 min (linear ablation rate: 0.00279 mm/s) and, after ablation, is in situ converted into a porous carbon aerogel, with the EMI shielding effectiveness jumping to 92.8 dB (X-band) and the strength reaching 8.62 MPa, thereby achieving a functional synergy transition from elastic thermal protection to high-efficiency electromagnetic shielding (Figure 10b) [93].

In summary, hierarchically porous structures, three-dimensional continuous conductive networks, and abundant heterogeneous interfaces serve as ideal microscopic topological configurations for achieving high-performance EMI shielding.

### 5.4. High-Performance Electrochemical Energy Storage

Phenolic-derived carbon aerogels also exhibit significant advantages in enabling fast ion transport and efficient charge storage. Through heteroatom doping and activation strategies, their electrochemical performance can be further enhanced, thereby delivering excellent rate capability and long-term cycling stability in supercapacitors and lithium/sodium-ion batteries [94,95,96]. In the context of pure carbon systems, Chang et al. used ethanol as the solvent and HCl as the catalyst to prepare, via a hydrothermal method combined with ambient-pressure drying, a carbon aerogel featuring a “bead-necklace”-like network structure (specific surface area: 759 m^2^/g). This material delivered a specific capacitance of 138.33 F/g in 6 M KOH at a scan rate of 5 mV/s. Upon further introduction of graphene oxide as a template to construct a lamellar-oriented structure, the specific capacitance increased to 150.00 F/g (an 8.4% enhancement), confirming the positive regulatory effect of ordered microstructures on charge-storage efficiency [97]. Zhao et al., by contrast, focused on intrinsic chemical modification by introducing boric acid into a phenolic resin precursor to form B–O crosslinking bonds. The resulting doped aerogel achieved a mass retention of 63.5% at 1000 °C (a 5.5% improvement over the undoped sample), and the optimised BCA-0.5 sample exhibited a high specific capacitance of 338.93 F/g at a current density of 2 A/g. This remarkable enhancement is primarily attributed to boron-doping-induced reductions in electrical resistance, enhanced ion-diffusion kinetics, and an increased number of electrochemically active sites, highlighting the order-of-magnitude gain in capacitive performance afforded by atomic-scale doping [98]. Building on this foundation, Cao et al. extended their research towards the synergistic integration of mechanical and electrochemical functionalities. They utilised aluminosilicate ceramic fibers to guide the oriented growth of phenolic aerogel particles along the fibers, forming a three-dimensional network that effectively suppressed volume shrinkage during drying and enhanced the mechanical integrity of the material. When the phenolic/curing-agent mass ratio was 8:1, the resulting composite delivered a discharge capacity of 330 mAh/g as a lithium-battery anode. This result is comparable to the typical specific capacity of commercial graphite, representing a valuable step toward validating the feasibility of applying phenolic aerogel-derived carbon materials in energy storage [99].

In summary, as shown in Table 3, phenolic aerogels, leveraging their intrinsic “lightweight–porous–thermally resistant” characteristics, are expanding their application scope from traditional thermal protection to diverse domains such as heterogeneous catalysis and lightweight composites, demonstrating a clear trend toward multifunctional integration.

## 6. Conclusions and Outlook

Phenolic aerogels, owing to their combination of low density, high porosity, high char yield, excellent thermal insulation, and favorable designability, have become a major research focus in high-performance thermal protection and functional porous materials. This review systematically surveys recent advances in the sol–gel preparation of phenolic aerogels, covering microstructure regulation, drying techniques, functional modification, and engineering applications. Through precise control over acid/base catalytic kinetics, phase-separation behavior, and nucleation–growth processes, directed construction of the nanoscale framework, pore-size distribution, and network connectivity can be achieved. The development of supercritical drying, freeze-drying, and ambient-pressure drying has further improved pore-structure retention, with ambient-pressure drying showing promising industrial prospects owing to its low cost and ease of scale-up. Although supercritical drying (SCD) offers solvent-recovery advantages at the laboratory scale, the electricity consumption and cooling requirements of industrial-scale, large-volume autoclaves still render its overall energy consumption higher than that of ambient-pressure processes; in contrast, while ambient-pressure drying (APD) reduces capital investment, it often entails extensive organic solvent exchange and surface modification steps, and the volatile organic compound (VOC) emissions associated with these steps should not be overlooked. Concurrently, organic–inorganic hybridization, fiber reinforcement, and synergistic modification with nanofillers have effectively overcome the bottlenecks in mechanical properties, high-temperature resistance, and oxidation resistance, driving the expansion of applications into aerospace thermal protection, electromagnetic interference shielding, environmental remediation, energy storage, and catalysis. Overall, the development of phenolic aerogels is shifting from single-property optimization toward cross-scale structural regulation and multifunctional integrated design.

Although phenolic aerogels exhibit excellent performance at the laboratory scale, their engineering application remains constrained by theoretical gaps, including an unclear understanding of the coupled mechanisms governing the sol–gel process, phase separation, and drying, as well as a lack of cross-scale structural evolution theories and in-situ characterization tools. At the engineering manufacturing level, skeleton shrinkage and pore-structure degradation during ambient-pressure drying limit the fabrication of large-sized products (e.g., >500 mm) and are further constrained by critical bottlenecks such as reproducibility, scalability, equipment and energy consumption, process stability, and the translation of laboratory-scale performance to practical service conditions. In addition, although bio-based monomers have demonstrated their feasibility as partial substitutes for traditional precursors, achieving a balanced trade-off among performance, cost, raw-material stability, and scalable processing remains a major challenge on the path toward large-scale industrial application. Future research should focus on low-cost, green, and scalable preparation technologies and establish a quantitative engineering indicator system that includes acceptable shrinkage, solvent recovery rate, unit cost, and long-term service life. Concurrently, these efforts should be integrated with artificial intelligence-assisted design and digital manufacturing and should incorporate high-entropy ceramics, ultra-high-temperature ceramics, and bio-inspired structures so as to develop a new generation of phenolic aerogel systems that are highly reliable, multifunctional, and intelligent.

## Figures and Tables

**Table 1:** Supercritical parameters of commonly used drying media [16,55].

Solvent	Supercritical Temperature (°C)	Supercritical Pressure (MPa)	Critical Density (g/cm^3^)
H_2_O	374.1	22.1	0.32
Ethanol	243.0	6.3	0.28
Benzene	289.5	4.9	0.32
CO_2_	31.1	7.3	0.47
Isopropyl alcohol	235.1	4.6	0.27
Acetone	235.0	4.7	0.28

**Table 3:** Comparison of properties and performance of phenolic aerogels and their derived carbon aerogels.

System	Hardener	Catalyst	Drying Method	Density (g/cm^3^)	Thermal Conductivity (W/(m·K))	Compressive Strength (MPa)	Linear Ablation Rate (mm/s)	Ref.
PF, quartz fiber felt	HMTA	-	atmospheric drying	0.27–0.28	0.061–0.064	2.8–5.7(50%)	-	[35]
RF, carbon fiber felt	-	CF_3_COOH	atmospheric drying	RF Aerogel 0.452–0.608 Carbon aerogel 0.296–0.576	RF Aerogel 0.133–0.250 Carbon aerogel 0.119–0.302	RF Aerogel 2.83–13.86 (10%) Carbon Aerogel 0.50–3.44 (10%)	0.045–0.081 (1.5 MW/m^2^, 30 s)	[27]
PF, quartz/carbon fiber felt	HMTA	HMTA	vacuum drying	0.23–0.27	0.052–0.068	1.08–4.23 (4.5%)	-	[38]
PF, quartz fiber felt	HMTA	-	atmospheric drying	0.508–0.510	0.102–0.135	-	0.032–0.042 (2.0 MW/m^2^, 60 s)	[47]
PF, carbon aerogel	HMTA	HMTA	supercritical CO_2_ drying, carburization, N_2_, 600–1000 °C, 1 h	0.203	0.048	1.94 (~3%)	-	[53]
RF, PAN fiber, carbon aerogel	-	Na_2_CO_3_	supercritical CO_2_ drying, vacuum, 1000 °C, 1 h	0.247	PAN-based C/CAs0.041~0.735	1.22 (10%)	-	[54]
RF	-	sodium acetate	Freeze-drying	0.09	0.039	0.024 (15%)	-	[33]
PF, carbon fiber felt	HMTA	HMTA	vacuum drying	0.43–0.70	0.131–0.229	16.50–28.92 (40%)	0.0160 (2 MW/m^2^, 60 s)	[83]

## Data Availability

No new data were created or analyzed in this study. Data sharing is not applicable to this article.
